# Association of SNP3 polymorphism in the apolipoprotein A-V gene with plasma triglyceride level in Tunisian type 2 diabetes

**DOI:** 10.1186/1476-511X-4-1

**Published:** 2005-01-06

**Authors:** Raja Chaaba, Nebil Attia, Sonia Hammami, Maha Smaoui, Sylvia Mahjoub, Mohamed Hammami, Ahmed Slaheddine Masmoudi

**Affiliations:** 1 Biochemistry laboratory, UR 08–39, Faculty of Medicine MONASTIR, Tunisia; 2 Department of Internal Medicine, C H U MONASTIR, Tunisia

## Abstract

**Background:**

Apolipoprotein A-V (Apo A-V) gene has recently been identified as a new apolipoprotein involved in triglyceride metabolism. A single nucleotide polymorphism (SNP3) located in the gene promoter (-1131) was associated with triglyceride variation in healthy subjects. In type 2 diabetes the triglyceride level increased compared to healthy subjects. Hypertriglyceridemia is a risk factor for coronary artery disease. We aimed to examine the interaction between SNP3 and lipid profile and coronary artery disease (CAD) in Tunisian type 2 diabetic patients.

**Results:**

The genotype frequencies of T/T, T/C and C/C were 0.74, 0.23 and 0.03 respectively in non diabetic subjects, 0.71, 0.25 and 0.04 respectively in type 2 diabetic patients. Triglyceride level was higher in heterozygous genotype (-1131 T/C) of apo A-V (p = 0.024). Heterozygous genotype is more frequent in high triglyceride group (40.9%) than in low triglyceride group (18.8%) ; p = 0.011. Despite the relation between CAD and hypertriglyceridemia the SNP 3 was not associated with CAD.

**Conclusion:**

In type 2 diabetic patients SNP3 is associated with triglyceride level, however there was no association between SNP3 and coronary artery disease.

## Background

Dyslipidemia in type 2 diabetes are most frequently characterized by elevation of total serum triglycerides, of very low density lipoprotein-triglyceride (VLDL-TG) and low level of high density lipoprotein-cholesterol (HDL-C) [1]. Hypertriglyceridemia is an independent risk factor for coronary artery disease (CAD) in type 2 diabetes [2]. Triglyceridemia is modulated by environmental and genetic factors. A new identified gene associated with triglyceride level was the gene encoding for apo A-V located at the chromosome 11 (11q23), in the vicinity of apoA-I/C-III/A-IV cluster [3]. Studies on transgenic mice overexpressing human apo A-V showed a decreased level of triglyceride, whereas knock-out mice showed an increased level of triglyceride [3]. These results prove the regulator effect of apo A-V on triglyceride metabolism. Moreover apo A-V regulates levels of circulating triglyceride and cholesterol [4]. Four neighboring single nucleotide polymorphisms (SNP1 to SNP4) within apo A-V were identified by Pennachio et al [3]. The first three of the SNPs (SNPs1–3) were in significant linkage disequilibrium suggesting the existence of a common haplotype in apo A-V gene. The minor allele of each SNP was associated with high triglyceride level. In other study, the SNP3 (T/C polymorphism) was also associated with HDL-C concentration [5]. This suggests that genetic variability of the apo A-V gene is likely to also have an impact on the lipid profile of type 2 diabetic patients, but reports on the subjects are few [6].

We have addressed the issue to examine the interaction between SNP3 and lipid profile and coronary artery disease (CAD) in type 2 diabetic patients compared to controls in Tunisian population.

## Results

### Description of the participating groups

Type 2 diabetic patients have a BMI values more important than non diabetic subjects. Whereas the WHR in lower in controls. Males and smokers are more frequent in patients with CAD (Table 1). To compare lipid parameters, we take into consideration the lipid lowering drugs use, then patients who are taking lipid lowering drugs were excluded (Table 2). Plasma total cholesterol and total triglyceride concentrations were significantly higher in type 2 diabetic patients than in the non diabetic patients. Subjects with CAD had lower concentration of HDL-C and higher concentration of triglyceride as compared to those without CAD (Table 2).

**Table 1:** Clinical characteristics of the four studied groups

	**Diabetes- CAD-**	**Diabetes+ CAD-**	**Diabetes+ CAD+**	**Diabetes- CAD+**	**Diabetes vs non Diabetes**	**CAD vs non CAD**
Number	99	78	74	57		

Sex (% of men)	50.5	49.4	75.4	84.2	ns	< 0.001
Age (years)	52.9 ± 8.7	51.5 ± 7.3	55.7 ± 6.8	59.0 ± 6.8	ns	< 0.001
BMI (Kg/m^2^)	27.6 ± 4.0	28.8 ± 4.6	28.1 ± 3.8	26.6 ± 4.8	0.029	ns
Smokers (%)	42	53	86	80	0.003	< 0.001
SBP (mmHg)	12.5 ± 1.3	13.2 ± 1.4	13.0 ± 1.8	12.4 ± 2.1	0.001	ns
DBP (mmHg)	7.5 ± 0.7	7.8 ± 0.9	7.6 ± 1.0	7.1 ± 1.1	0.006	0.018
WHR	0.82 ± 0.09	0.92 ± 0.07	0.95 ± 0.07	0.92 ± 0.06	ns	0.016
Glucose (mmol/l)	5.2 ± 0.4	10.4 ± 3.4	11.7 ± 3.5	6.8 ± 3.6	< 0.001	< 0.001
HbA1c (%)	4.5 ± 0.9	10.4 ± 3.4	9.4 ± 2.4	11.3 ± 4.1	0.009	< 0.001
Diabetes duration (years)		6.8 ± 5.2	7.9 ± 6.1			
Lipid lowering drug use (%)	11.1	0	16.2	70.2		

SBP : systolic blood pressure

DBP : diastolic blood pressure

**Table 2:** lipid profiles of the four studied groups

	**Diabetes- CAD-**	**Diabetes+ CAD-**	**Diabetes+ CAD+**	**Diabetes- CAD+**	**Diabetes vs non Diabetes**	**CAD vs non CAD**
Number	88	78	62	17		
Cholesterol (mmol/l)	4.70 ± 1.22	5.16 ± 1.14	5.13 ± 1.12	4.06 ± 1.22	< 0.001	ns
HDL-C (mmol/l)	0.94 ± 0.24	0.96 ± 0.38	0.79 ± 0.22	0.72 ± 0.21	ns	< 0.001
Total TG (mmol/l)	1.39 ± 0.75	2.11 ± 1.48	2.27 ± 1.77	2.81 ± 2.53	< 0.001	< 0.001

### Heterozygous genotype had the high triglyceride level

According to SNP3 of the apo A-V gene, variation of lipid parameters in diabetic or non diabetic patients are shown in Table 3. Non diabetic subjects having the heterozygous genotype (T/C) showed an increased triglyceride level and decreased HDL-C concentration. However these variations were not significant. In type 2 diabetic patients, triglyceride level increased significantly in C/T genotype in association with non significant decrease in HDL-C concentration. The genotype frequencies of T/T, T/C and C/C were 0.74, 0.23 and 0.03 respectively in non diabetic subjects, 0.71, 0.25 and 0.04 respectively in type 2 diabetic patients. The SNP3 was shown to be in Hardy-Weinberg equilibrium. It is clear that there was no difference in genotype distribution between diabetic and non diabetic subjects. The type 2 diabetic population was classified further into those with high and low triglyceride concentration (cut point 2.2 mmol/l which was more than 90 percentile level in the healthy population). The SNP3 frequencies for T/T, T/C and C/C genotypes in the low triglyceride groups were 77.1 %, 18.8 % and 4.2 % respectively, and those in the high triglyceride group were 59.1 %, 40.9 % and 0 % respectively. The difference in genotype frequencies between low and high triglyceride groups were significant (p = 0.011) (Table 4).

**Table 3:** Clinical Characteristics and lipid profile according to SNP3 of apo A-V in type 2 diabetic and in non diabetic subjects

	**Type 2 Diabetes**		**Non Diabetes**	
Characteristics	**Genotypes**			

	**TT**	**TC**	**TT**	**TC**
Number	100	36	76	25
Sex (% men)	61	50	55.8	60
Smokers (%)	67.3	81.3	44.4	46.2
Age (years)	52.8 ± 7.6	54.8 ± 6.2	53.3 ± 8.8	51.5 ± 7.5
BMI (Kg/m^2^)	28.5 ± 4.3	28.7 ± 3.9	27.3 ± 3.0	28.1 ± 5.0
WHR	0.94 ± 0.06	0.93 ± 0.08	0.93 ± 0.08	0.9 ± 0.08
Cholesterol (mmol/l)	5.17 ± 1.15	5.17 ± 1.07	4.61 ± 1.32	4.53 ± 0.99
HDL-C (mmol/l)	0.90 ± 0.32	0.83 ± 0.34	0.92 ± 0.25	0.83 ± 0.26
Total TG (mmol/l)	2.05 ± 1.61	2.62 ± 1.59*	1.6 ± 1.42	1.75 ± 1.07

* significant difference between TT and TC genotypes (p = 0.016).

**Table 4:** Distribution of different apo A-V genotypes in type 2 diabetes between low and high triglyceride groups

	**Low triglyceride group ≤ 2.2 mmol/l**		**High triglyceride group > 2.2 mmol/l**	
Polymorphism	**Number**	**%**	**Number**	**%**
T/T	74	77.1	26	59.1
T/C	18	18.8	18	40.9
C/C	4	4.2	nd	nd
Total	96	100	44	100

nd : not detected

X2 = 8.962, p = 0.011, degree of freedom = 2.

### No association between SNP3 and CAD

To investigate the relation of SNP3 polymorphism with coronary artery disease we studied the association in all subjects (those who are taking lipid lowering drugs are included). There was no association between coronary artery disease and SNP3 either in non diabetic subjects or in type 2 diabetic patients (Table 5).

**Table 5:** Association between SNP3 of apo A-V and risk for coronary artery disease in diabetic and non diabetic subjects

	**Genotypes**	**CAD-**	**CAD +**	
Diabetes-	T/T	74 (74.7)	41 (71.9)	P = 0.514 OR = 1.29 (0.6–2.77)
	T/C	21 (21.1)	15 (26.3)	
	C/C	4 (4.0)	1 (1.8)	

Diabetes+	T/T	56 (71.8)	52 (70.3)	P = 0.717 OR = 0.87 (0.41–1.83)
	T/C	21 (26.9)	17 (23.0)	
	C/C	1 (1.3)	5 (6.7)	

OR was calculated using T/T and T/C genotypes only.

## Discussion

Elevated serum lipid levels are an important risk factor for atherosclerosis. Both environmental and genetic factors contribute to variability in serum lipid levels [5]. In this study, we choose the apo A-V gene as a genetic factor that predispose to elevated triglyceride level. The dynamic interfacial properties of apo A-V are consistent with the hypothesis that apo A-V impedes triglyceride particle assembly [7]. Thus, the effect of apo A-V on triglyceride level can be attributed to the intracellularly function of apo A-V to modulate hepatic VLDL synthesis and/or secretion. Also apo A-V can lower plasma triglyceride by activating the lipoprotein lipase (LPL)[8]. In our study we showed that SNP3 was significantly associated with hypertriglyceridemia especially in type 2 diabetic patients. This finding confirmed the idea that the effect of this SNP on triglyceride metabolism was not influenced by ethnic background [9]. However, it has been reported that the major and minor allele frequencies differed between populations such as : 0.06, 0.09 and 0.34 for the C allele in UK, Caucasian and Japanese respectively [10,3,9]. In our population, the minor allele frequency (0.13) is almost the same as in Caucasian [3] but lower than in Japanese population [9]. However, the triglyceride level is higher in Tunisian than in Japanese population. This paradoxical observation confirms that triglyceride level is influenced by environmental factors and other genetic factors (apo CIII...).

In type 2 diabetes the triglyceride level is increased, which is due to multiple factors related to insulin and carbohydrate metabolism, LPL activity, CETP activity... The absence of an unusual allele frequency of SNP3 in type 2 diabetic patients compared to non diabetic subjects, in spite of triglyceride level variation, shows that there is no association between apo A-V SNP3 polymorphism and the presence or absence of diabetes. Diabetic homozygous for the major allele are more frequent in low triglyceride group, showing that SNP3 is associated with triglyceride variation in type 2 diabetic patients. Contrary to Esteve et al., who have reported no significant difference in triglyceride concentration with the apo A-V polymorphism in type 2 diabetic patients [6], our results showed, then, that SNP3 is associated with triglyceride level.

The relationship between SNP3 and coronary artery disease is under discussion. The LOCAT study showed that there is no association between SNP3 and progression of coronary heart disease [11]. Our results find no association between SNP3 of apo A-V and development of coronary artery disease either in diabetic patients or in non diabetic subjects. This suggests that the high triglyceride level in T/C genotype alone was not a good discriminator of coronary heart disease. In contrast ; Szalai et al. showed an association between SNP3 and an increased risk for severe coronary artery disease [12]. Identifying genetic and environmental factors that influence plasma lipid levels represents a key step towards developing strategies for preventing and treating CAD. Usually, in the case of type 2 diabetes, patients are taking lipid lowering drugs in order to ameliorate their lipid profile, namely lower triglyceride level and increase HDL-C concentration. Fibrates represent a commonly used therapy for lowering plasma triglyceride, its mechanism of action involves the activation of the nuclear receptor peroxisome proliferator activated receptor alpha (PPARα). The apo A-V is a highly responsive PPAR target gene [13]. While SNP3 is located in promoter region, it should be interesting to study the interaction between this polymorphism and lipid lowering drugs response in different population.

## Conclusion

In summary, the apo A-V SNP3 is associated with triglyceride level in Tunisian type 2 diabetic patients. However, this SNP is unlikely to be associated with the presence of diabetes. Although SNP3 is associated with hypertrigyceridemia, there was no relationship between this polymorphism and coronary artery disease. Further investigations were needed to determine the effect of SNP3 on lipid lowering drug response.

## Methods

### Subjects

Three hundred and eight subjects, aged 45–70 years, participated in this study. They belong to four groups. The first group contained 74 type 2 diabetic patients with CAD, among whom 12 are taking lipid lowering drugs. The second group contained 78 type 2 diabetic patients without CAD, all of them did not take lipid lowering drugs. The third group contained 57 patients with CAD and without diabetes, 70% of the patients are taking lipid lowering drugs. The last group contained 99 controls without diabetes nor CAD, 11 subjects are taking lipid lowering drugs. The clinical and biological characteristics of each group are summarized in Table 1.

All participants were recruited in the departments of internal medicine and cardiology in Monastir university hospital. Written or verbal informed consent were obtained from all patients and controls before the study. All written informed consent were not possible because the majority of eligible subjects in our study were illiterate. The diagnosis of diabetes was based on a previous history of diabetes according to American Diabetes Association criteria [14]. The patients with CAD were defined as clinical history of stable angina pectoris, previous acute coronary syndromes with or without ST segment elevation. This CAD was confirmed by coronary angiography. Exclusion criteria were taking insulin, having a renal or liver failure or thyroid disease, alcohol consumption before 3 days or less, Body Mass Index (BMI) more than 35 Kg/m^2 ^and glycosylated hemoglobin (Hb A1c) more than 12%. Post menopausal women had no hormone replacement therapy. BMI was calculated using the formula : weight (Kg)/height^2 ^(m^2^). Obesity was defined as BMI>30 kg/m^2^. Waist-to-hip ratio (WHR) was calculated from measurements of the waist circumference taken at the mid point between umbilicus and xiphoid and hip circumference, at the widest point around the hips, respectively. Blood samples were drawn after subjects had fasted overnight (12 hours) into tubes containing EDTA. Plasma was immediately separated by centrifugation.

### Laboratory Analysis

After DNA extraction, the single nucleotide polymorphism SNP3 within the apo A-V gene (-1311 T/C) was determined by PCR-RFLP analysis using MseI restriction endonuclease as described previously [3]. Plasma glucose, glycosylated haemoglobin (HbA1c), lipids and lipoproteins were determined as described by Smaoui et al. [15].

### Statistical Analyses

Data management and statistical analysis were performed using SPSS 10.0 software. Results are summarized as mean ± SD. Since triglyceride levels were not normally distributed, logarithmic transformation of triglyceride concentration was performed before the statistical analysis. Student's test was used to compare continuous variables and Chi square (χ^2^) test was used to examine distribution of categorical variables. A value of p < 0.05 was considered significant.

## List of abbreviations

Apo : apolipoprotein

BMI : Body Mass Index

CAD : Coronary Artery Disease

HDL : High Density Lipoproteins

HDL-C : HDL cholesterol

PCR-RFLP : Polymerase Chain Reaction-Restriction Fragment Length Polymorphism

SNP : Single Nucleotide Polymorphism

TG : Triglyceride

VLDL : Very Low Density Lipoproteins

WHR : Waist-to-Hip Ratio

## Authors' contributions

R.Ch and N.A: carried out the molecular studies, participated in the design of the study and drafted the manuscript ; M.S : carried out the biochemical essay ; S.H and Sy.M : interested in the clinical aspect ; M.H : conceived of the study, and participated in its design and coordination and helped to draft the manuscript and revised it critically for important intellectual content and have given final approval of the version to be published; MS.M : revised the article critically for important intellectual content and have given final approval of the version to be published.
